# Snail Upregulates Transcription of FN, LEF, COX2, and COL1A1 in Hepatocellular Carcinoma: A General Model Established for Snail to Transactivate Mesenchymal Genes

**DOI:** 10.3390/cells10092202

**Published:** 2021-08-26

**Authors:** Tam Minh Ly, Yen-Cheng Chen, Ming-Che Lee, Chi-Tan Hu, Chuan-Chu Cheng, Hsin-Hou Chang, Ren-In You, Wen-Sheng Wu

**Affiliations:** 1 Institute of Medical Sciences, Tzu Chi University, Hualien 970, Taiwan; 105353116@gms.tcu.edu.tw; 2 Division of General Surgery, Department of Surgery, Hualien Tzu Chi Hospital, Buddhist Tzu Chi Medical Foundation, Hualien 970, Taiwan; yccmdsurg@gmail.com; 3 Division of General Surgery, Department of Surgery, Hualien Tzu Chi Hospital, Buddhist Tzu Chi Medical Foundation and School of Medicine, Tzu Chi University, Hualien 970, Taiwan; mclee1229@mail.tcu.edu.tw; 4 Division of Gastroenterology, Department of Medicine, Research Centre for Hepatology, Hualien Tzu Chi Hospital, Buddhist Tzu Chi Medical Foundation, Hualien 970, Taiwan; chitan.hu@msa.hinet.net; 5 Department of Laboratory Medicine and Biotechnology, College of Medicine, Tzu Chi University, Hualien 970, Taiwan; cordiallove@yahoo.com.tw; 6 Department of Molecular Biology and Human Genetics, Tzu Chi University, Hualien 970, Taiwan; hhchang@mail.tcu.edu.tw

**Keywords:** snail, fibronectin, lymphoid enhancer-binding factor, cyclooxygenase 2, collagen type alpha I, transcription

## Abstract

SNA is one of the essential EMT transcriptional factors capable of suppressing epithelial maker while upregulating mesenchymal markers. However, the mechanisms for SNA to transactivate mesenchymal markers was not well elucidated. Recently, we demonstrated that SNA collaborates with EGR1 and SP1 to directly upregulate MMP9 and ZEB1. Remarkably, a SNA-binding motif (TCACA) upstream of EGR/SP1 overlapping region on promoters was identified. Herein, we examined whether four other mesenchymal markers, lymphoid enhancer-binding factor (LEF), fibronectin (FN), cyclooxygenase 2 (COX2), and collagen type alpha I (COL1A1) are upregulated by SNA in a similar fashion. Expectedly, SNA is essential for expression of these mesenchymal genes. By deletion mapping and site directed mutagenesis coupled with dual luciferase promoter assay, SNA-binding motif and EGR1/SP1 overlapping region are required for TPA-induced transcription of LEF, FN, COX2 and COL1A1. Consistently, TPA induced binding of SNA and EGR1/SP1 on relevant promoter regions of these mesenchymal genes using ChIP and EMSA. Thus far, we found six of the mesenchymal genes are transcriptionally upregulated by SNA in the same fashion. Moreover, comprehensive screening revealed similar sequence architectures on promoter regions of other SNA-upregulated mesenchymal markers, suggesting that a general model for SNA-upregulated mesenchymal genes can be established.

## 1. Introduction

The poor prognosis of hepatocellular carcinoma (HCC) is due to frequent metastasis occurring via complicated processes, including epithelial mesenchymal transition (EMT), migration, and invasion of primary tumor [1]. Among the metastatic transcription factors, Snail (SNA) was not only overexpressed in HCC and linked with a poor prognosis [2,3] but may also accelerate EMT, invasion, and metastasis of HCC [1,3,4,5,6]. Additionally, SNA was regarded as a promising therapeutic target for preventing the progression of various tumors, including HCC [7]. SNA can be regulated by receptor tyrosine kinase (RTK) signaling triggered by many metastatic factors, including hepatocyte growth factor (HGF) and epidermal growth factor (EGF). While SNA directly represses epithelial markers like E-cadherin, it also upregulates the mesenchymal markers, including vimentin, fibronectin, and matrix metalloproteinases (MMP-9) in HCC [8,9,10]. To complete its EMT program, SNA also upregulates other mesenchymal transcription factors such as Twist and zinc finger E-box binding homeobox 1 (ZEB1) in HCC [11,12]. The mechanisms for SNA to suppress epithelial markers such as E-cadherin are well elucidated. SNA can bind to the E-boxes (5’-CACCTG) on the E-cadherin promoter and recruit various epigenetic machinery such as histone deacetylase, hence repressing E-cadherin expression [13,14]. In contrast, the detailed mechanisms for SNA to upregulate the transcription of mesenchymal markers are far less clarified. Previous studies indicated that SNA activated transcription of MMP9 and ZEB1 indirectly via regulation of other transcriptional factors such as Twist, Ets-1, and SP1 or microRNAs in different contexts [15,16]. However, our recent report demonstrated SNA collaborates with EGR1 and SP1 to directly upregulate MMP9 and ZEB1 stimulated by the phorbol ester tumor promoter 12-O-tetradecanoyl-phorbol 13-acetate (TPA) in HepG2. Importantly, a specific putative SNA binding motif “TCACA”, required for activating their promoters was identified [17]. This is similar to those observed in the transcription of cyclin-dependent kinase 4/6 (CDK4/6), p15INK4b, a cell cycle-related gene upregulated by SNA [18]. Intriguingly, after examining sequences of the other known SNA upregulated mesenchymal markers, including vimentin, TWIST1, vitronectin, α2 smooth muscle actin (ACTA2), fibronectin (FN), and lymphoid enhancer-binding factor (LEF), we found similar sequence architecture, i.e., the SNA motif TCACA coupled with neighboring EGR1 and SP1 region, also exists in their promoters around 300–2000 bp upstream of the translational initiation sites. Thus, it is tempting to investigate whether SNA can directly activate transcriptional upregulation of other EMT-related genes involved in HCC progression such as FN [19], LEF [20], COX2 [21,22,23], and COL1A1 [24,25,26] in a similar fashion as observed in MMP9 and ZEB1. By gene expression and promoter analysis coupled with protein–DNA interaction assay in HCC, we did find that FN, LEF, COX2, and COL1A1 were transcriptionally upregulated by SNA via binding to the proposed SNA binding motifs, which are close to the EGR-1/SP1 overlapping regions.

## 2. Materials and Methods

### 2.1. Cell Culture

The cultured conditions for HCC340 and HepG2 cells were the same as described in our previous report [17]. HCC340 is a patient-derived cell line established on January 2014 from parts of HCC tissue obtained from surgery with patient’s consents, which have been approved by Buddhist Tzu Chi General Hospital Research Ethic Committee (IRB 101–62). The isolation of HCC cell line was performed according to the methodology described in our previous report [27]. Briefly, HCC tissues were pretreated with collagenase and cultivated on the mitomycin C-treated NIH3T3 feeder layer for 4–6 passages. Homogenous HCC cell populations were obtained and the sustained proliferation ability (over 20 passages) was tested. Cell lines obtained from this method were authenticated by short tandem repeat (STR) assay, analyzing the characteristic of microsatellite patterns of the patients’ genomes (unpublished results).

### 2.2. Chemicals and Antibody

Tetradecanoyl phorbol acetate (TPA) was purchased from Sigma-Aldrich (Poole, UK). The monoclonal Snail (C15D3) antibody; and the antibodies against EGR1, SP1, MMP9, ZEB1, LEF1, Fibronectin, COX2, COL1A1, Histone H3, and GAPDH were purchased from Cell Signaling (Beverly, MA, USA) and Santa Cruz Biotechnology (Santa Cruz, CA, USA), respectively. The snail expression plasmid, p-Snail, driven by CMV promoter within the p-cDNA3 vector, was a gift from Dr. Cheng K.K., Tzu Chi University.

### 2.3. Constructions of Various Promoter Plasmids for Deletion Mapping

The promoter regions in the full-length promoter plasmids of LEF, FN, COX2, and COL1A1 were amplified from the genomic sequence of each gene. The PCR products were ligated into pGL3 vector (Promega, Madison, WI, USA). The promoter plasmids of deletion constructs were derived from each full-length promoter by double digestion with various restriction enzymes, followed by filling in the restriction site overhangs by Klenow enzyme. Subsequently, the digested DNA fragments were ligated into pGL3 vector.

### 2.4. Site-Directed Mutagenesis on Promoters

The full-length promoter plasmids of LEF, FN, COX2, and COL1A1 were used as templates for site-directed mutagenesis using a GeneEditor in vitro site-directed mutagenesis system (Promega, Madison, WI, USA) to obtain various mutant promoters according to the manufacturer’s protocol. The bases changed in the site-directed mutagenesis for the proposed Snail binding motif (TCACA), and the EGR1/SP1 overlapping binding region were the same as those described in our previous report [17,18].

### 2.5. Transwell and Wound Healing Migration Assays

Transwell and wound healing migration assays were performed as described in previous report [8,24].

### 2.6. Dual-Luciferase Promoter Assay

Dual-Luciferase promoter assay was performed according to the protocol of manufacture (Promega, Madison, WI, USA).

### 2.7. Chromatin Immunoprecipitation (ChIP) Assay

ChIP assay was performed as described in our previous Reports [17,18]. Supplemental Figure S1 showed the schematic maps of the PCR fragments amplified for the ChIP assay of SNA, EGR1, and SP1 on indicated promoter regions of LEF, FN, COX2, and COL1A1. The primer sequences for ChIP assay are demonstrated in Table 1.

### 2.8. RT-PCR and Quantitative RT-PCR

RT-PCR was performed as described in our previous report [18]; the primers used for RT-PCR and real-time PCR were shown in Table 2.

### 2.9. Electrophoresis Mobility Shift Assay (EMSA)

EMSA was performed as described in our previous report [18]. Nuclear–cytoplasmic protein fractions used for EMSA were collected using the NE-PER Nuclear and Cytoplasmic Extraction Reagents kit (Thermo Fisher Scientific, Waltham, MA, USA) according to the manufacturer’s protocol. The sequence of the biotin-labeled probe (25 bp) and un-labeled wild type and mutant competitors derived from indicated promoters are shown in Table 3.

### 2.10. Western Blot

Western Blot was performed as described in our previous Reports [17,18].

### 2.11. shRNA Technology

shRNA Technology was performed as described in our previous Reports [18]. The sequences of shRNA fragments targeting different regions of Snail were the same as those used in our previous study [18].

### 2.12. Statistical Analysis

Data were analyzed using Student’s *t*-test in Excel. All the quantitative studies were performed at least in triplicate, as appropriate. Statistical significance between groups were indicated by * *p* < 0.05 and ** *p* < 0.001.

## 3. Results

### 3.1. SNA Is Essential for Constitutive and TPA-Induced Gene Expression of FN, LEF, COX2, and COL1A1 in HepG2 and HCC340 Cells

**Figure 1:**
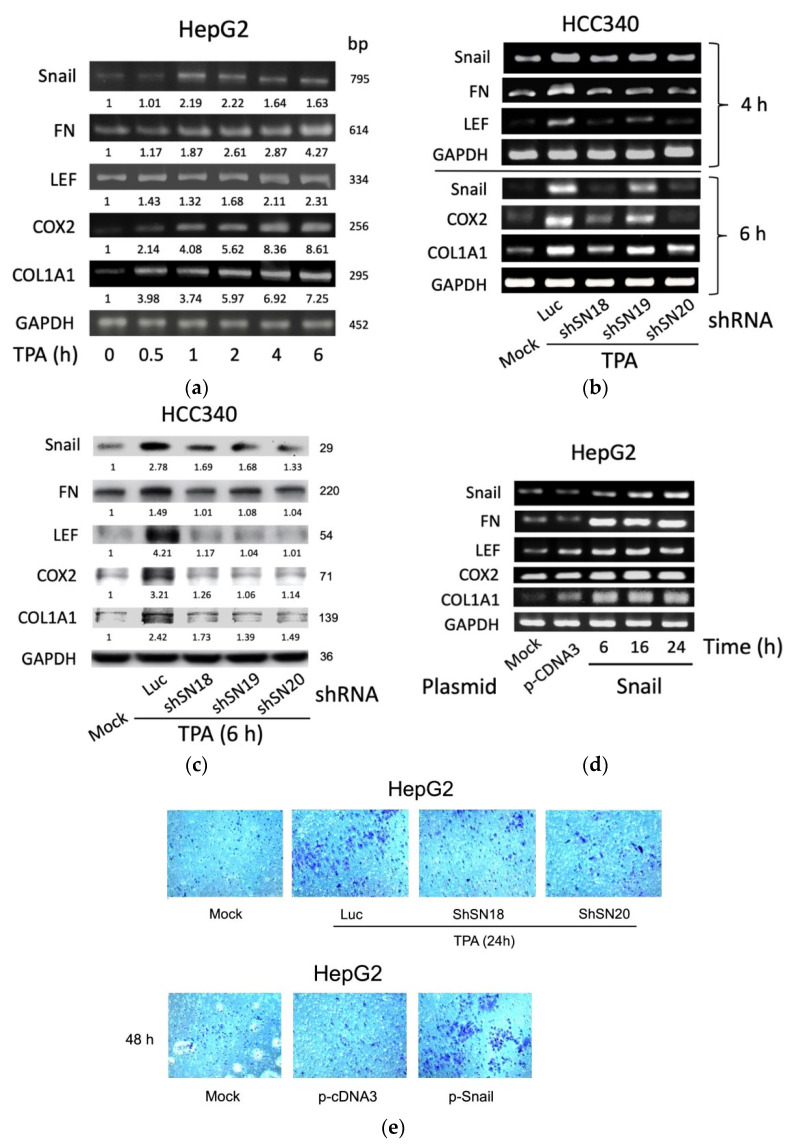
SNA is essential for constitutive and required TPA-induced gene expression of FN, LEF, COX2, and COL1A1 and cell migration of HCCs. HepG2 cells were treated with 50 nM TPA for the indicated time (**a**), HCC340 cells were transfected with luciferase shRNA (Luc) or different shRNAs of SNA for 24 h followed treated with TPA for 4 h ((**b**), upper panel), 6 h ((**b**), lower panel and (**c**)) and 24 h ((**e**), upper panel), HepG2 were transfected with SNA expressing plasmid for the indicated time (**d**) ((**e**), lower panel), using p-CDNA3 as a control vector. RT/PCRs (**a**,**b**,**d**), Western blot (**c**) of the indicated genes and transwell migration assay (**e**) were performed. In (**a**–**d**), GAPDH is used as an internal control. The data shown are representative of three reproducible results. In (**a**,**c**), the numbers indicated below each band are averaged relative intensities (Coefficient of Variation: 7–10%), taking time zero (**a**) or MOCK group (**c**) as 1.0.

Initially, by RT-PCR, we found TPA elevated mRNA of SNA by 3.7-fold at 1 h and declined until 6 h, whereas it significantly increased mRNA of FN, LEF, and COX2 by 1.5–2-fold within 1–2 h, which was further increased to 2.5–4.8-fold until 6 h in HepG2, one of the conventional HCC cell lines (Figure 1a). Also, TPA increased mRNA of COL1A1 within 0.5 h by 2.0-fold, which was gradually increased until 6 h by 4.8-fold (Figure 1a). The time courses of the TPA-induced expression of these genes were also quantitatively analyzed in HCC340, a patient-derived HCC cell lines used for studying the SNA-upregulated MMP9 and ZEB1 transcription [17]. As shown in quantitative RT-PCR analysis (Supplemental Figure S2a), the maximal induction of SNA mRNA by TPA was observed at 4 h, whereas those of FN, LEF, COX2, and COL1A1 were observed at 6 h. Consistently, Western blot demonstrated TPA maximally elevated FN, LEF, COX2, and COL1A1 proteins during 6–8 h by 1.8–2.9-fold whereas TPA induced dramatic elevation of SNA earlier at 4 h and gradually declined at 8 h (Supplemental Figure S2b). Together, this implicated SNA is an upstream regulator of FN, LEF, COX2, and COL1A1. To address this issue, we further investigated whether depletion of SNA may influence the expression of these genes. In a conventional RT-PCR analysis (Figure 1b, upper panel), TPA-induced elevation of FN and LEF mRNA were found to be attenuated in HCC340 transfected with shRNAs of SNA, shSN18, and shSN20 by 65–80% at 4 h, compared with those in the cells transfected with the control Luciferase (Luc) shRNA. However, another SNA shSN19 exhibited less preventive effect. Quantitative-RT-PCR revealed similar results using HepG2 (Supplemental Figure S3a, left panel). Also, TPA-induced elevation of COX2 and COL1A1 at 6 h were greatly reduced in HCC340 transfected with shSN18 and shSN20 by 65–90%, whereas those in the cells transfected with shSN19 had only 35–55% reduction (Figure 1b, lower panel and Supplemental Figure S3a, right panel). Consistently, the knockdown efficiencies of shSN18 and shSN20 were about 70–95% while that of shSN19 was only 30–40% in HCC340 (Figure 1b). Moreover, Western blot demonstrated 60–90% reduction of protein level of these genes induced by TPA for 6 h in HCC340 transfected with the aforementioned SNA shRNAs (Figure 1c). On the contrary, overexpression of SNA for 6–24 h elevated FN, LEF, COX2, and COL1A1 mRNAs by 3–10-fold in HepG2 using conventional RT-PCR (Figure 1d) and in both HepG2 and HCC340 using quantitative RT-PCR (Supplemental Figure S3b). This is consistent with the result demonstrating that overexpression of SNA for 24 h increased FN, LEF, COX2, and COL1A1 protein by 1.5–2.5-fold in HepG2 (Supplemental Figure S3c). Since the role of SNA in upregulating these mesenchymal genes for EMT and cell migration is well established, we examined whether alteration of SNA can influence the motility of both HCCs. As shown in the transwell migration assay (Figure 1e, upper panel), transfection of shSN18 and shSN20 for 48 h significantly attenuated TPA-induced cell migration of HepG2 for 24 h by about 60–70% compared with the control luciferase shRNA. On the other hand, overexpression of SNA dramatically increased migration of HepG2 compared with the p-c-DNA3 vector (Figure 1e, lower panel). In addition, overexpression of SNA significantly increased migration of HCC340 and HepG2 in wound healing migration assays (Supplemental Figure S4). Collectively, SNA is essential for gene expression of FN, LEF, COX2, and COL1A1 required for cell migration of HCCs.

### 3.2. SNA Is Essential for Constitutive and TPA-Induced Transcriptional Activation of FN, LEF, COX2, and COL1A1 in HCC

**Figure 2:**
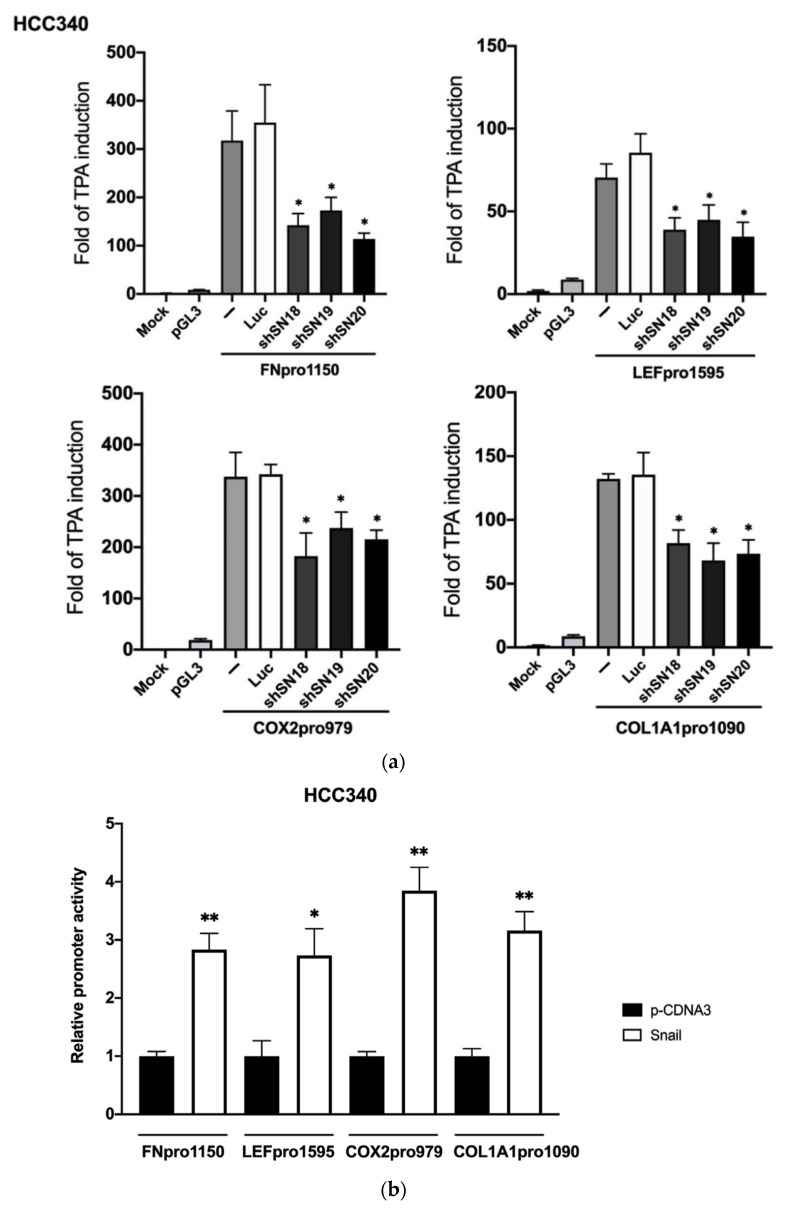
SNA is essential for constitutive and TPA-induced promoter activation of FN, LEF, COX2, and COL1A1. (**a**) HCC340 cells were untransfected (MOCK), transfected with PGL3 vector or full-length promoter of FN (upper left panel), LEF (upper right panel), COX2 (lower left panel), or COL1A1 (lower right panel) coupled with none (−), luciferase (Luc) or various SNA shRNAs as indicated for 16 h. Subsequently, the cells were treated with TPA for 12 h. MOCK is the nontransfected and no TPA-treated sample. Dual-luciferase activity was performed. For each full-length promoter, the fold of TPA induction was calculated by comparing the activity of each sample with the data of MOCK. (**b**) HCC340 were transfected with none (MOCK), p-cDNA3, or SNA expression plasmid for 24 h, followed by transfection of full-length promoter of FN, LEF, COX2, or COL1A1 for 16 h. Dual-luciferase was performed. For each full-length promoter, relative activity was calculated, taking the data of p-cDNA3 (**b**) as 1.0. In (**a**,**b**), (*, **) represent the statistically significant difference (*p* < 0.05, *p* < 0.001, N = 3) between the indicated samples and Luciferase shRNA (**a**) or p-cDNA3 group (**b**).

We further examined whether TPA can induce promoter activation of FN, LEF, COX2, and COL1A1 in a SNA-dependent manner. To this end, full-length promoter plasmid of FN (FNpro1150), LEF (LEFpro1595), COX2 (COX2pro979), and COL1A1 (COL1A1pro1090) were constructed by inserting the promoter fragment containing 1150 bp and 1595 bp, 979 bp and 1090 bp, respectively, upstream of the translation start site of each promoter into PGL3 vector. As shown in Figure 2a, TPA elevated promoter activation of FNpro1150 (upper left panel), LEFpro1595 (upper right panel), COX2pro979 (lower left panel), and COL1A1pro1090 (lower right panel) by 310-, 60-, 330- and 170-fold, respectively, in HCC340. Transfection of SNA shRNAs, including shSN18, shSN19 or shSN20 suppressed the TPA-induced promoter activation of FNpro1150, LEF pro1595, COX2pro979, and COL1A1pro1090 by 45–70% compared with that of the control (Luciferase, Luc) shRNA group. In addition, TPA-induced promoter activation of FNpro1150 and LEFpro1595 can be suppressed by aforementioned SNA shRNAs by 60–70% in HepG2 (Supplemental Figure S5). Moreover, overexpression of SNA increased constitutive promoter activation of FNpro1150, LEFpro1595, COX2pro979, and COL1A1pro1090 by 2–2.5-fold in HCC340 (Figure 2b) and HepG2 (data not shown). Collectively, SNA is essential for constitutive and TPA-induced transcriptional activation of FN, LEF, COX2, and COL1A1 in HCC.

### 3.3. Deletion Mapping Identified SNA Motif and EGR1/SP1 Overlapping Regions within TPA-Response Element on FN, LEF, COX2, and COL1A1 Promoters

**Figure 3:**
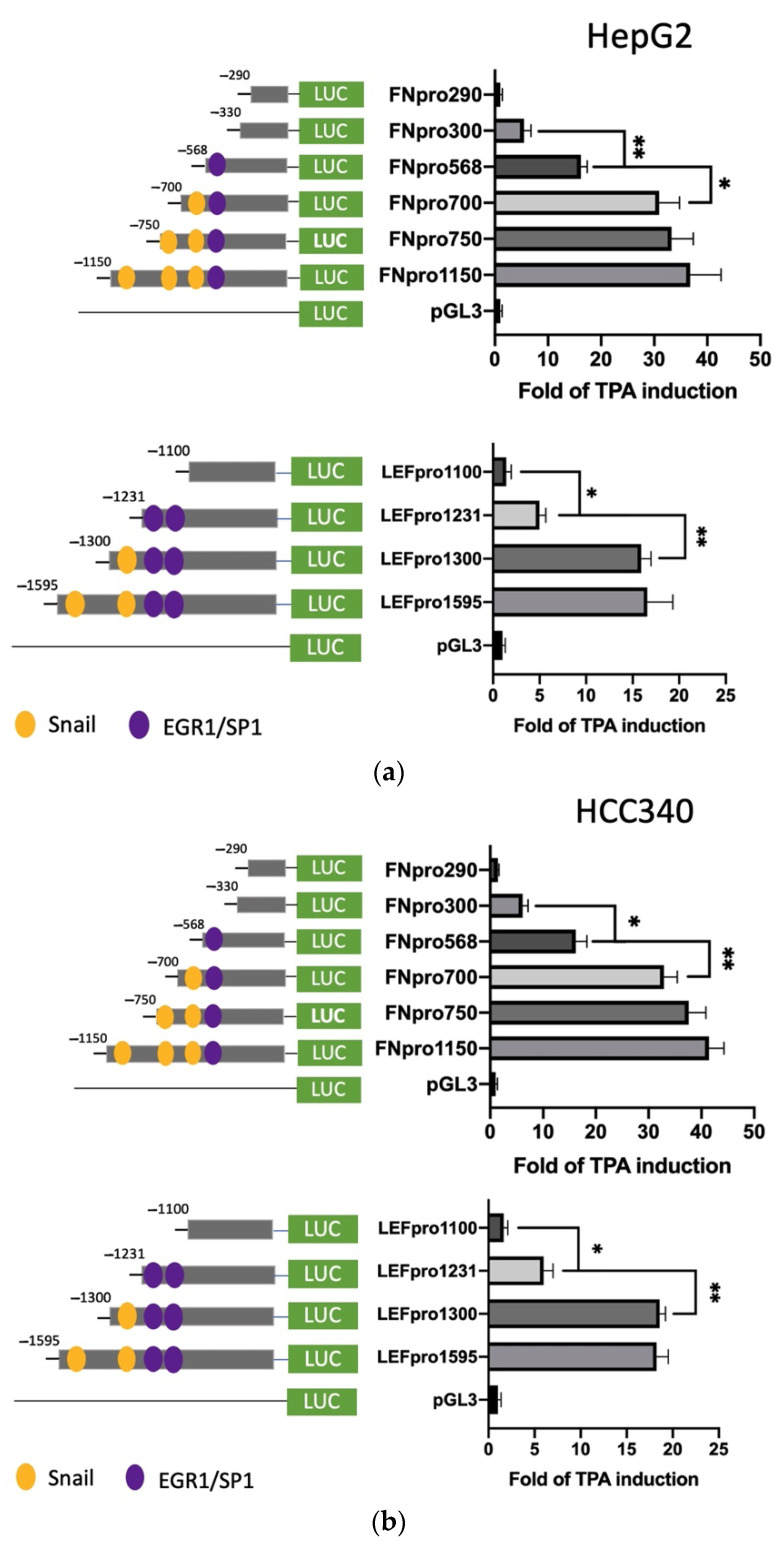

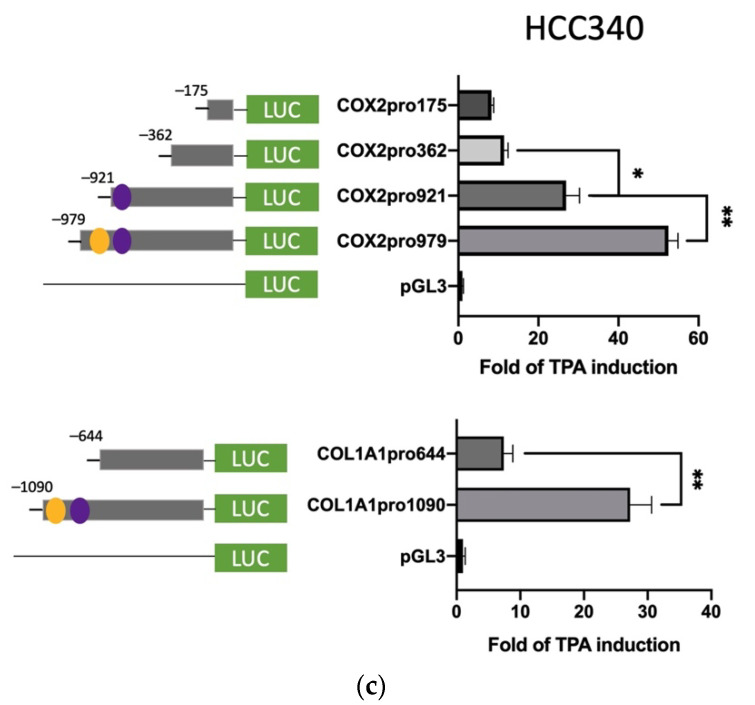
Deletion mapping for identifying TPA-responsive element on FN, LEF, COX2, and COL1A1 promoter. HepG2 (**a**) and HCC340 (**b**,**c**) cells were transfected with PGL3, the full-length promoter of FN (FNpro1150) (a and b, upper panel), LEF (LEFpro1595) ((**a**,**b**), lower panel), COX2 (COX2pro921) and COL1A1 (COL1A1pro1090) ((**c**), upper and lower panel, respectively) or the promoter constructs deleted from 5′ end of each of the full-length promoters as indicated, followed by TPA treatment for 12 h (**a**–**c**). Dual-luciferase was performed. Relative fold of TPA-induced luciferase activity was calculated, taking the data of pGL3 group as 1.0. In each figure, (*, **) represent the statistically significant difference (*p* < 0.05, *p* < 0.001, N = 5) between the indicated promoter fragments.

Further, we sought to identify the TPA-responsive element on promoters of FN and LEF, COX2, and COL1A1 and examined whether it contained the proposed SNA binding region “TCACA” and putative EGR1/SP1 overlapping motif, as described previously [17]. According to the Genomatix software, there are three and two proposed SNA binding regions, respectively, locating on the promoter of FN (−1166 to −547 bp upstream of the translation start site) (Supplemental Figure S6a) and LEF (−1617 to −1211 bp upstream of the translation start site) (Supplemental Figure S6b). To examine whether any of these SNA binding motifs were required for promoter activation of both FN and LEF, deletion mapping analysis using 5′ end truncated mutants including FNpro750, FNpro700, and FNpro568 (excluding 1, 2, and 3 SNA region, respectively, from 5′ end on full-length FN promoter) (Figure 3a, upper left panel), LEFpro1300 and LEFpro1231 (excluding 1, and 2 SNA regions, respectively, from 5′ end on full-length LEF promoter) (Figure 3a, lower left panel) were performed. As demonstrated in Figure 3a (upper right panel) and Figure 3b (upper right panel), the TPA-induced promoter activation of FNpro750 and FNpro700, the FN mutants lacking one and two distal SNA motifs, respectively, decreased only 10–20% compared with FNpro1150 in both HepG2 (Figure 3a) and HCC340 (Figure 3b). Strikingly, FNpro568, the mutant lacking all 3 SNA regions, exhibited a dramatic reduction (by 68%) of TPA-induced promoter activation compared with FNpro1150 in both HCCs. Similarly, the TPA-induced promoter activation of LEFpro1300, the LEF mutants lacking one SNA motif, exhibited minor difference (by 10–20%) compared with LEF pro1595, whereas LEFpro1231, the mutant lacking both SNA motifs exhibited a more significant reduction (by 70–80%) of TPA-induced promoter activation than those of LEFpro1595 and LEFpro1300 in HepG2 (Figure 3a, lower right panel) and HCC340 (Figure 3b, lower right panel). Together, these implied that the most proximal (3′) SNA motifs are required for TPA-induced promoter activation of both FN and LEF. Interestingly, the TPA-responsive SNA motifs on both promoters are close to a EGR1/SP1 (E/S) overlapping region, which was known to be required for TPA-induced, SNA-mediated MMP9, and ZEB1 promoter activation [17]. Thus, it is very probable that the E/S overlapping regions close to the candidate SNA site on both promoters are also involved in SNA-dependent transcriptional upregulation in FN and LEF. Indeed, FNpro330 and LEFpro1100, which lack E/S overlapping and all the SNA regions, showed a further decrease (by 80–90%) of TPA-induced promoter activation than those of the FNpro568 and LEFpro1231 that still contained E/S overlapping region in both HCCs (Figure 3a,b). We further investigated whether the transcription regulations of COX2 and COL1A1 are also the same as FN and LEF. There is one proposed SNA binding region and a downstream EGR1/SP1, located −1010 to −502 bp and −1135 to −934 bp, respectively, upstream of the translation start site on the promoter of COX2 and COL1A1 (Figure S6c,d). Thus, we constructed 5′ end truncated mutants of the full-length promoter, COX2pro921, lacking the SNA binding motif (Figure 3c, upper left panel). Also, COX2pro362 and COX2pro175, with deletion of both SNA binding motif and the downstream EGR1/SP1 overlapping regions, were included (Figure 3c, upper left panel). On the other hand, COL1A1pro644 without SNA binding motif and the downstream EGR1/SP1 overlapping region were obtained (Figure 3c, lower left panel). As shown in Figure 3c (upper right panel), the TPA-induced promoter activation of COX2pro921 and COX2pro362 decreased by 50 and 80%, respectively, compared with full length COX2pro979 in HCC340. On the other hand, the TPA-induced promoter activation of COL1A1pro644 was lower than that of full-length promoter COL1A1pro1090 by 60% (Figure 3c, lower right panel). Collectively, these strongly suggest that the most proximal (3′) SNA region coupled with the adjacent E/S overlapping regions are essential for TPA-induced promoter activations of FN, LEF, COX2, and COL1A1.

### 3.4. Mutagenesis on Promoter Validated SNA Binding Motif and E/S Overlapping Region for Transcription of FN, LEF, COX2, and COL1A1

**Figure 4:**
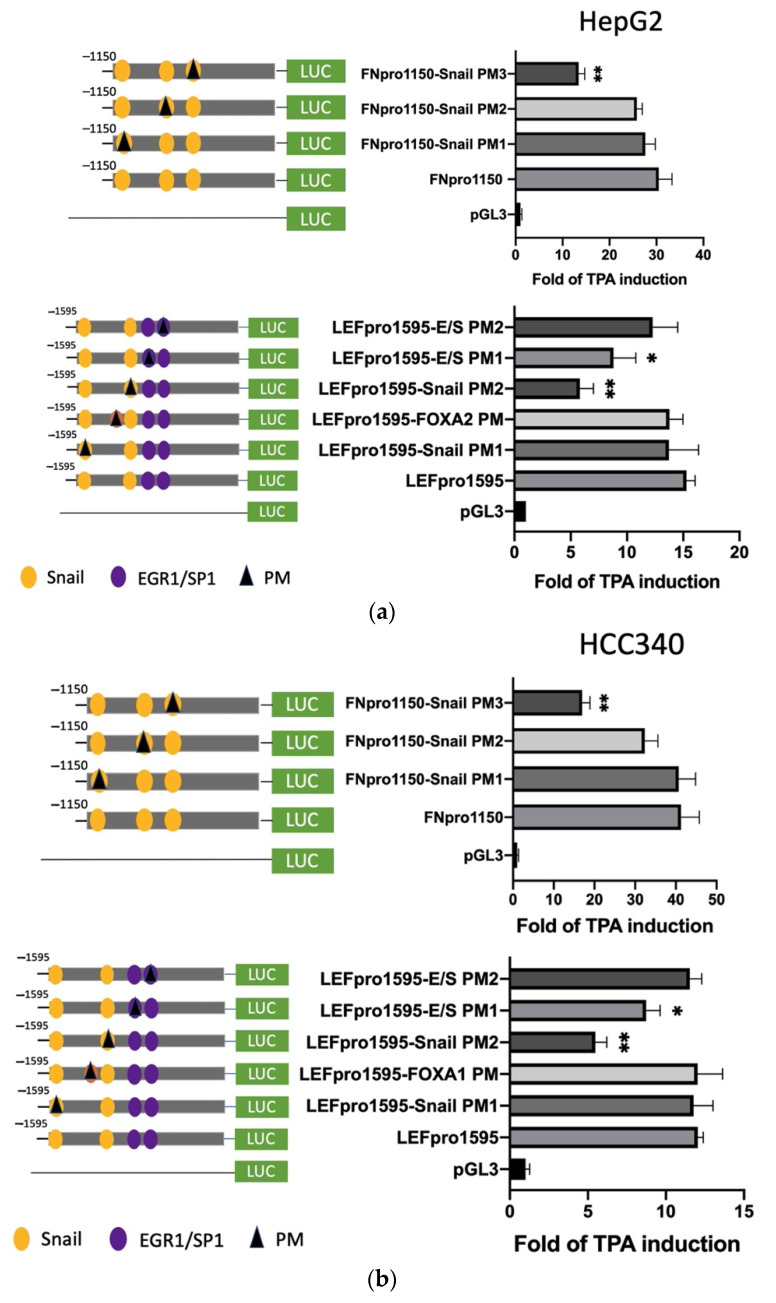

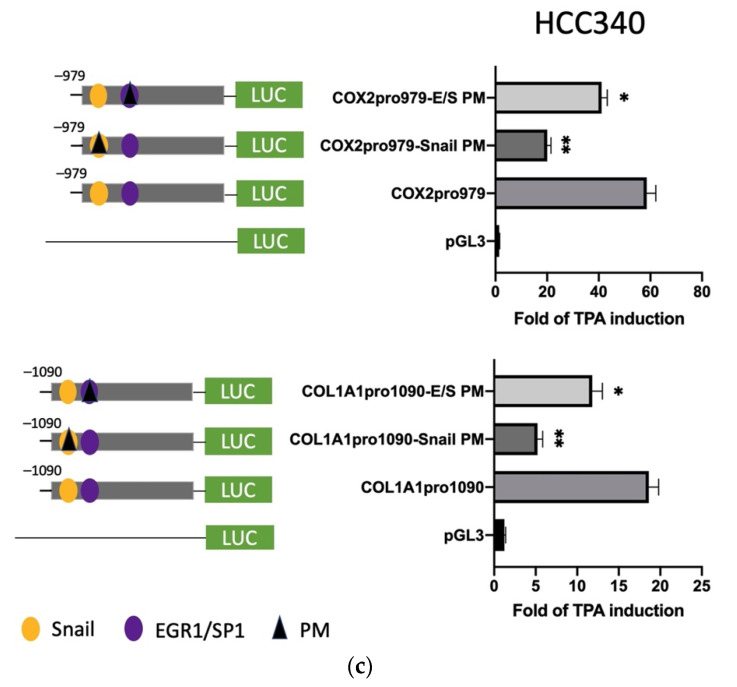
Site-directed mutagenesis for identifying TPA-responsive element on FN, LEF, COX2, and COL1A1 promoter. HepG2 (**a**) and HCC340 (**b**,**c**) were transfected with pGL3, the wild type FNpro1150, LEFpro1595 (**a**,**b**), COX2pro921, and COL1A1pro1090 (**c**) or site-directed mutated types of each promoter with altered SNA binding sites or EGR1/SP1 region, as indicated (PM 1, 2 or 3: point mutation 1, 2 or 3), followed by TPA treatment for 12 h. Dual-luciferase was performed. Relative fold of TPA-induced luciferase activity was calculated, taking the data of pGL3 group as 1.0. In each figure, (*, **) represent the statistically significant difference (*p* < 0.05, *p* < 0.001, N = 3) between the indicated mutant promoter group and the wild-type promoter.

To prove whether the candidate SNA binding motifs are required for transcription of the aforementioned mesenchymal genes, site-directed mutagenesis coupled with promoter analysis were performed. For FN, promoter mutants with the altered sequence (TCACA→TTGTA) at indicated SNA motifs (PM 1–3) were produced for analysis in both HepG2 (Figure 4a, upper left panel) and HCC340 (Figure 4b, upper left panel). Remarkably, FN promoter mutant with the altered sequence at the proximal (3′) SNA binding motif (PM3) exhibited a 60% decrease of TPA-induced promoter activation compared with that of the wild type FNpro1150 in both HepG2 (Figure 4a, upper right panel) and HCC340 (Figure 4b, upper right panel) at 12 h, whereas mutation of FN promoter at the middle SNA (PM2) and the distal (5′) SNA region (PM1) did not decrease the TPA-induced promoter activation. Similarly, LEF promoter mutant with the altered sequence at the proximal (3′) SNA (PM2) but not the distal (5′) SNA (PM1) (Figure 4a,b, lower left panel) exhibited a significant decrease (by 50–70%) of TPA-induced promoter activation than that of the wild type LEFpro1595 in both HepG2 (Figure 4a, lower right panel) and HCC340 (Figure 4b, lower right panel). Collectively, these results validated that the most proximal (3′) SNA binding regions on promoters of both FN and LEF are essential for TPA-induced promoter activation, consistent with the data obtained in deletion mapping analysis (Figure 3). In addition, mutation (CCCCGCCT→CCCTATCT) on the E/S region (−1195 to −1179 bp) of LEFpro1595 (LEFpro1595 E/S-PM1) close to the active SNA motif, but not the next (proximal) E/S region (−1148 to −1131 bp) (LEFpro1595 E/S-PM2) (Figure 4a,b, lower left panel) also attenuated the TPA-enhanced promoter activation of LEFpro1595 by 33% in HepG2 (Figure 4a, lower right panel) and HCC340 (Figure 4b, lower right panel). As a negative control, mutagenesis on the FOXA2 region close to the active SNA region has no effect on TPA-induced promoter activation of LEF (Figure 4b, lower panel). On the other hand, mutagenesis of SNA binding motif on COX2 and COL1A1 promoter decreased the TPA-induced promoter activation of the full length COX2pro979 and COL1A1pro1090 by 66 and 72%, respectively, whereas mutagenesis of EGR1/SP1 overlapping region attenuated the aforementioned full-length promoters by 30% (Figure 4c). Taken together, the proximal (3′) SNA region coupled with the adjacent E/S overlapping region are responsible for TPA-induced promoter activation of FN, LEF, COX2, and COL1A1.

### 3.5. ChIP Validated the Binding of Key Transcription Factors on Putative Regions In Vivo

**Figure 5:**
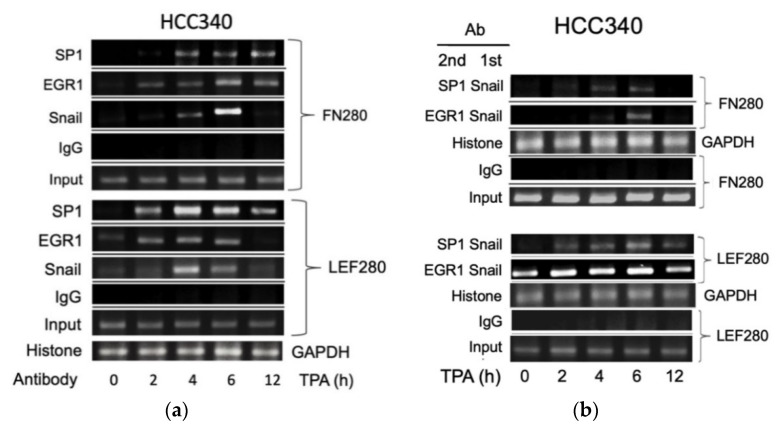

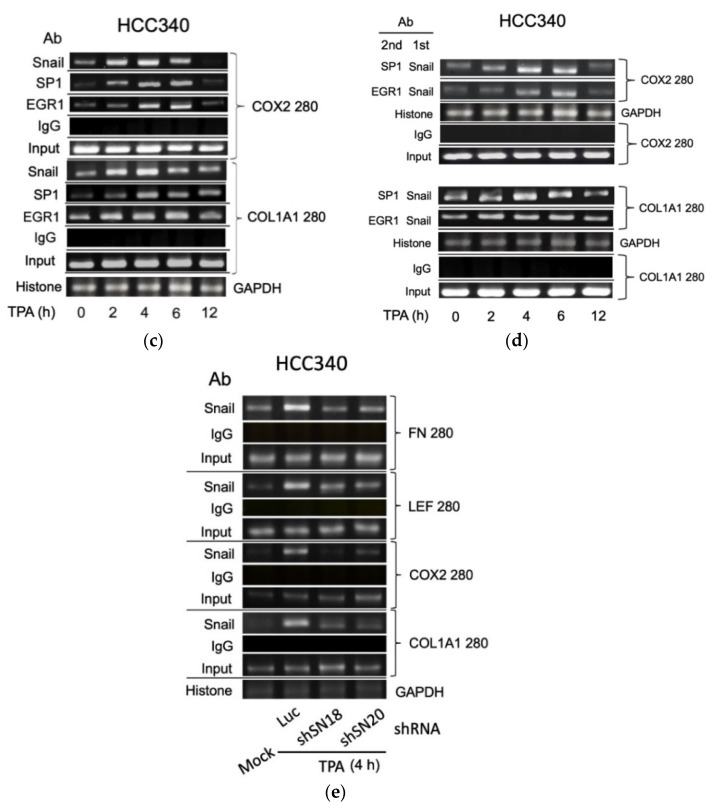
ChIP and double ChIP assay for TPA-induced binding of transcription factors on promoter of FN, LEF, COX2, and COL1A1. HCC340 cells were treated with TPA at the indicated time (**a**–**d**). HCC340 cells were transfected with indicated shRNA for 48 h, followed by treatment of TPA for 4 h (**e**). Single ChIP for binding an indicated transcription factor on indicated promoter fragment (**a**,**c**,**e**) and double ChIP for the association of indicated factors on indicated promoter fragments (**b**,**d**) were performed using conventional PCR. In (**a**–**e**), IgG was used as the antibody control and histone binding on GAPDH promoter as positive control. Input was for normalizing the amount of DNA loaded for ChIP. The data shown were representatives of two reproducible experiments.

Thus far, it appears that SNA coupled with EGR1/SP1 activate transcription of the aforementioned mesenchymal genes, whether the relevant transcriptional factors can bind to the indicated putative regions was examined within cells using ChIP assay. As shown in Figure 5a (upper panel), TPA can induce binding of SNA on a FN promoter fragment (FN280), containing sequences of the critical SNA coupled with EGR1/SP1 region, at 4 h, further increased at 6 h and returned to basal level at 12 h in HCC340. Also, TPA can induce binding of EGR1 on the same promoter fragment at 2–4 h, further increased at 6 h, and declined at 12 h. In addition, TPA can induce sustained binding of SP1 on the same fragment during 4–12 h (Figure 5a, upper panel). Similarly, TPA-induced binding of SNA, EGR1, and SP1 on the LEF promoter fragment (LEF280) containing the critical SNA motif coupled with EGR1/SP1 region begins at 2 h, further increased at 4 h and gradually declined during 6–12 h (Figure 5a, lower panel). Moreover, double ChIP assay validated the association of SNA with EGR1 (by 1st ChIP SNA, 2nd ChIP EGR1), and SNA with SP1 (by 1st ChIP SNA, 2nd ChIP SP1) on FN280 at 4 h, further increased at 6 h and declined at 12 h (Figure 5b, upper panel). Similarly, TPA induced association of SNA with EGR1, and SNA with SP1 at 2 h on LEF280, which were further increased at 4–6 h and declined at 12 h (Figure 5b, lower panel). Furthermore, the ChIP and double ChIP assays for binding the aforementioned transcriptional factors on both promoters were quantitatively analyzed by quantitative PCR. As shown in Supplemental Figure S7a (left panel), TPA can induce binding of SNA and EGR1 on FN280 by 4.2- and 8.8-fold at 4 h, which was further increased at 6 h by 17- and 20-fold and returned to 2 and 4-fold of the basal level at 12 h in HCC340, whereas binding of SP1 on FN280 can be induced at 2 h by 3.0-fold, which was increased to 8.0-fold at 4 h and sustained to 12 h. Similarly, TPA can induce binding of SNA, EGR1, and SP1 on LEF280 at 2 h by 1.5–2.0-fold, which were increased to 3.5-fold at 4 h, significantly decreased at 6 h, and returned to basal at 12 h (Supplemental Figure S7b, left panel). Moreover, double ChIP assay demonstrated a 5.5- and 8.5-fold increase in the association of (SNA with EGR1) and (SNA with SP1) on FN280, respectively, at 6 h of TPA treatment (Supplemental Figure S7a, right panel). Likewise, TPA can induce association of (SNA with EGR1) and (SNA with SP1) on LEF280 by 3.5- and 3.0-fold, respectively, at 6 h (Supplemental Figure S7b, right panel). In ChIP assays of COX2 and COL1A1, TPA can increase prominent binding (by about 2.8-fold) of SNA on a COX2 promoter fragment (COX2 280), containing sequences of the critical SNA coupled with EGR1/SP1 region at 2 h, which was further increased by about 3.8-fold at 4 h declined to 2.2-fold at 6 h and returned to basal level at 12 h in HCC340 (Figure 5c, upper panel). Also, TPA can induce maximal binding of EGR1 and SP1 on the same promoter fragment at 4–6 h which was declined at 12 h (Figure 5c, upper panel). Similarly, TPA-induced maximal binding of SNA on the COL1A1 promoter fragment (COL1A1 280) containing the critical SNA coupled with EGR1/SP1 region began at 4 h and gradually declined during 6–12 h (Figure 5c, lower panel). Besides, TPA can induce maximal binding of EGR1 and SP1 on the same promoter fragment at 4 and 6 h, respectively (Figure 5c, lower panel). Moreover, double ChIP assay validated the association of SNA with EGR1 (by 1st ChIP SNA, 2nd ChIP EGR1), and SNA with SP1 (by 1st ChIP SNA, 2nd ChIP SP1) on COX2 promoter fragment (COX2 280) induced by TPA at 4 h and 6 h and declined at 12 h (Figure 5d, upper panel). Similarly, TPA induced association of SNA with EGR1 and SNA with SP1 during 2–6 h on COL1A1 promoter fragment (COL1A1 280) (Figure 5d, lower panel). Expectedly, knockdown of SNA by shSN18 and shSN20 greatly suppressed the TPA-induced binding of SNA at 4 h on the promoter fragments containing SN binding motif of FN, LEF, COX2, and COL1A1 (Figure 5e).

### 3.6. EMSA Assay Validated the Binding of SNA on Putative Regions of FN, LEF, COX2, and COL1A1 Promoter In Vitro

**Figure 6:**
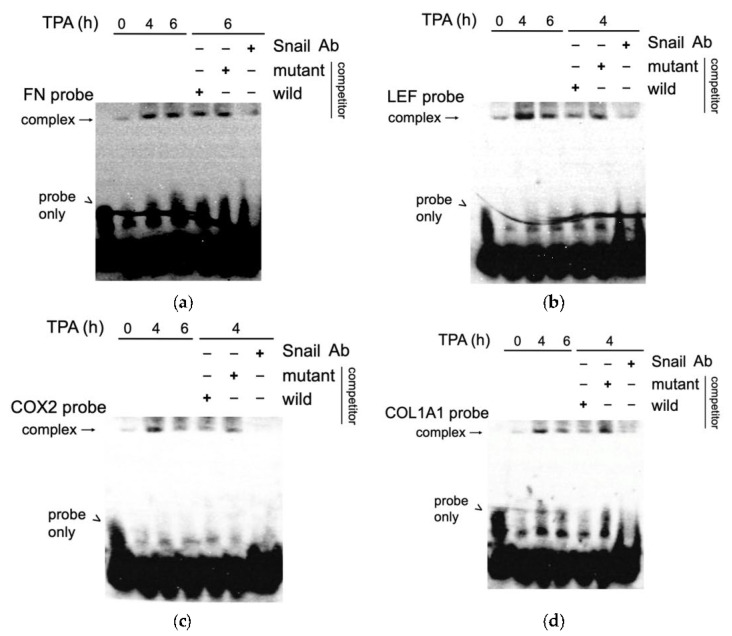
EMSA for TPA induced binding of SNA on putative regions of FN, LEF, COX2, and COL1A1 promoter. Nuclear extracts obtained from HCC340 treated with 50 nM TPA for the times indicated were used for EMSA for promoter fragment of FN (FN probe) (**a**), LEF (LEF probe) (**b**), COX2 (COX2 probe) (**c**), and COL1A1(COL1A1 probe) (**d**). Unlabeled wild-type or mutant competitors in 200X amount were included in the EMSA using nuclear extracts from HCC340 treated with TPA for 6 h (for FN probe) or 4 h (for LEF probe, COX2 probe, and COL1A1 probe). Lane 1 is the sample of probe only. For antibody inhibition, SNA antibody was preincubated with the nuclear extract from HCC340 treated with TPA for 6 h (for FN probe) and 4 h (for LEF probe, COX2 probe, and COL1A1 probe), followed by EMSA reaction. The data shown are representative of two reproducible experiments.

We further confirmed the binding of SNA on the aforementioned promoters by EMSA. Nuclear extract of HCC340 treated with TPA at different time points were incubated with biotin-labeled probe containing the SNA binding motif on FN, LEF, COX2, and COL1A1 promoter (FN probe, LEF probe, COX2 probe, and COL1A1 probe, respectively). As shown in Figure 6a, a weak DNA-protein complex derived from FN probe was observed in the time zero group, which was increased by TPA at 4 and 6 h by 6- and 5.2- fold, respectively. The addition of 200-fold of the unlabeled wild type but not the mutant type of probe reduced the amount of the complex at 6 h of TPA treatment by 50%. Furthermore, preincubating the EMSA mixture with the SNA antibody decreased the amount of DNA-protein complex, confirming that the SNA was protein-bound with the DNA probe. A similar phenomenon was also observed in EMSA of the DNA probe containing the critical SN1 binding motif on LEF (LEF probe), COX2 (COX2 probe), and COL1A1 (COL1A1-probe) promoter. As demonstrated in Figure 6b–d, TPA induced a prominent band shift of the complexes derived from the indicated promoter probes at 4 h, which was decreased at 6 h. As expected, the TPA-induced band shift of the DNA-protein complex at 4 h can be completed by 200-fold of unlabeled wild type but not mutant type probe (Figure 6b–d). Moreover, preincubation of the EMSA mixture with SNA antibody resulted in reducing the indicated complexes (Figure 6b–d), validating that SNA was protein-bound with the aforementioned promoter probes.

## 4. Discussion

In the past decades, it appeared that the mechanisms by which SNA upregulates the expression of mesenchymal genes were more complicated than it downregulates epithelial markers. As demonstrated in this and our previous [17] reports, SNA upregulates mesenchymal genes such as MMP9, ZEB1, FN, LEF, COX2, and COL1A1 by directly binding on a consensus motif to activate target promoters in HCC. However, many previous studies have indicated that SNA also upregulates mesenchymal markers in an indirect fashion. For example, SNA mediated the TGFβ-induced MMP9 activation by promoting the binding of SP-1/Ets-1 and nuclear factor kappaB (NFkappaB) to the proximal and distal promoter regions, respectively, in MDCK cells [15]. Also, SNA upregulated transcription of ZEB1 by elevating gene expression of Twist and triggering the nuclear translocation of Ets [16]. Recently, it is emerging that SNA-regulated gene expression involves a negative feedback circuit established by the cross-talk between SNA and non-coding RNAs, including miRNAs, long non-coding RNAs, and circular RNAs [28,29]. Collectively, there are multifaceted mechanisms for SNA to upregulate target gene expression.

Previously, the role of SNA in upregulating transcription of FN, LEF, COX2, and COL1A1 has been reported in different contexts. SNA can mediate TGFβ-triggered LEF expression for EMT in MDCKII cells [30]. Overexpression of SNA elevated FN to trigger EMT of retinal pigment epithelial (RPE) cells involved in proliferative vitreoretinopathy [31]. Also, SNA mediated FGF2-dependent expression of FN involved in the endothelial-mesenchymal transition leading to retrocorneal membrane formation and blindness [32]. SNA, on the other hand, has been demonstrated to increase the production of proinflammatory cytokines and cyclooxygenase-2 (COX2), both of which have been correlated with malignancy previously [28]. Also, increased Snail1 in FECD cells was responsible for an increased responsiveness to TGF-β present in the aqueous humor and excessive production of type I collagen [33]. Moreover, Snail contributes to pancreatic tumor development by promoting fibrotic reaction through increased collagen expression under TGFβ signaling [34]. However, whether SNA directly transactivates the aforementioned mesenchymal genes has not been established yet. One previous study demonstrated that SNA coupled with the p65 subunit of NF-κB and PARP1 bound to the FN1 promoter at proximal (−236/+72) region to activate FN1 transcription in melanoma cells [35]. Thus, the possibility that SNA collaborates with other transcriptional factors to directly upregulate transcription of mesenchymal genes via binding to specific regions different from what we have identified (i.e., TCACA) cannot be excluded.

Thus far, we have demonstrated six of the mesenchymal genes, FN, LEF, MMP9, ZEB1, COX2, and COL1A1, are transcriptionally upregulated by SNA in the same manner, i.e., by direct binding of SNA coupled with EGR1/SP1 on their putative regions. To predict whether this could be a general model, we screened the mesenchymal genes that can be upregulated by SNA from PubMed and examined whether they contain the aforementioned SNA binding motif (TCACA) coupled with a downstream EGR1/SP1 overlapping region in the gene bank. In addition to what we have investigated, five of the well-known mesenchymal genes such as vimentin, vitronectin, α-SMA, N-cadherin, and TWIST1 were identified (Table 4). All of them not only can be upregulated by SNA involved in tumor progression but also contain the candidate SNA and EGR/SP1 overlapping regions on their promoter (Table 4). It is worthy of investigating whether they can be transcriptionally upregulated by SNA in the same way as described in this and a previous study [17].

In conclusion, we proposed a general model for SNA to upregulate transcription of mesenchymal genes via collaboration with EGR1/SP1 in HCC. Remarkably, a specific putative SNA binding motif “TCACA” close to the EGR1/SP1 overlapping region, required for activating their promoters, was identified. In the future, this model will be validated after more of the candidate mesenchymal genes are investigated in HCC and other cancers.

## Figures and Tables

**Table 1:** Primers used for the ChIP assays.

Gene	Primer Sequence	Product Size
FN280	F: 5′GGG AAG GGG GAG CGT CTT3′	280 bp
	R: 5′CCC GCC CCA CCC CAC CCG3′	
LEF280	F: 5′CTC GCC AAG TTG CCT GAT3′	280 bp
	R: 5′CTC CCC ACT GCT TCT CCT3′	
COX2 280	F: 5′GTC CAT CAG AAG GCA GGA AAC3′	280 bp
	R: 5′CTA TAT GCA GCA CAT ACA TAC3′	
COL1A1 280	F: 5′AGG GTC TCT AAG CAG CCC CTG3′	280 bp
	R: 5′GAT GGA GTG GGG AGG CTG AGG3′	
GADPH	F: 5′TAC TAG CGG TTT TAC GGG CG3′	166 bp
	R: 5′TCG AAC AGG AGG AGC AGA GAG CGA3′	

**Table 2:** Primers used for RT-PCR and real time PCR in gene expression analysis.

Gene	Primer Sequence	Product Size
Snail or	F: 5′AAGC TTCC ATGG CGCG CTCT TTCC TCGT CAGG AAGC CC3′	795 bp
	R: 5′GGAT CCTC AGCG GGGA CATC CTGA GCAG CCGG ACTC TTG3′	
	F: 5′TCCA GAGT TTAC CCTT CCAG CA3′	218 bp
	R: 5′CTTT CCCA CTGT CCTC ATCT G3′	
FN or	F: 5′GTGC CTGG GCAA CGCA3′	614 bp
	R: 5′CCCG ACCC TGAC CGAA G3′	
	F: 5′AAG GAG AAG ACC GGA CCA AT3′	314 bp
	R: 5′GGC TTG ATG GTT CTC TGG AT3′	
LEF	F: 5′TGG CAG CCC TAT TTC AGT TT3′	334 bp
	R: 5′CAA AGG CTG TGC TTG CTT TT3′	
COX2	F: 5′CGG TGA AAC TCT GGC TAG ACA G3′	256 bp
	R: 5′GCA AAC CGT AGA TGC TCA GGG A3′	
COL1A1	F: 5′TCT GCG ACA ACG GCA AGG TG3′	295 bp
	R: 5′GAC GCC GGT GGT TTC TTG GT3′	
GADPH or	F: 5′ACC ACA GTC CAT GCC ATC AC3′	452 bp
	R: 5′TCC ACC ACC CTG TTG CTG TA3′	
	F: 5′ACGG ATTT GGTC GTAT TGGG3′	215 bp
	R: 5′TGAT TTTG GAGG GATC TCGC3′	

**Table 3:** EMSA probe of DNA–Snail binding reaction.

**Biotin-Labeled Oligonucleotide Probe**		
FN	F: FN-585~-560	5′CCCCT TCGCT **TCACA** CAAGT CCAGC3′
	R: FN-585~-560	5′GCTGG ACTTG **TGTGA** AGCGA AGGGG3′
LEF	F: LEF-1297~-1272	5′CACAC CACAC **TCACA** CACCC CAAAA3′
	R: LEF-1297~-1272	5′TTTTG GGGTG **TGTGA** GTGTG GTGTG3′
COX2	F: COX2-975~-950	5′CCCGT GGAGC **TCACA** TTAAC TATTT3′
	R: COX2-975~-950	5′AAATA GTTAA **TGTGA** GCTCC ACGGG3′
COL1A1	F: COL1A1-1080~-1055	5′TCACC AATGA **TCACA** GGCCT CCCAC3′
	R: COL1A1-1080~-1055	5′GTGGG AGGCC **TGTGA** TCATT GGTGA3′
**Unlabeled Oligonucleotide Competition Probe**		
FN	F: FN-Snail wild	5′CCT TCGCT **TCACA** CAAGT CC3′
	R: FN-Snail wild	5′GG ACTTG **TGTGA** AGCGA AGG3′
	F: FN-Snail mutant	5′CCT TCGCT T**TGT**A CAAGT CC3′
	R: FN-Snail mutant	5′GG ACTTG T**ACA**A AGCGA AGG3′
LEF	F: LEF-Snail wild	5′CAC CACAC **TCACA** CACCC CA3′
	R: LEF-Snail wild	5′TG GGGTG **TGTGA** GTGTG GTG3′
	F: LEF-Snail mutant	5′CAC CACAC T**TGT**A CAAGT CC3′
	R: LEF-Snail mutant	5′TG GGGTG T**ACA**A GTGTG GTG3′
COX2	F: COX2-Snail wild	5′GTG GAGC **TCACA** TTAAC TAT3′
	R: COX2-Snail wild	5′ATA GTTAA **TGTGA** GCTC CAC3′
	F: COX2-Snail mutant	5′GTG GAGC T**TGT**A TTAAC TAT3′
	R: COX2-Snail mutant	5′ATA GTTAA T**ACA**A GCTC CAC3′
COL1A1	F: COL1A1-Snail wild	5′CCA ATGA **TCACA** GGCCT CCC3′
	R: COL1A1-Snail wild	5′GGG AGGCC **TGTGA** TCAT TGG3′
	F: COL1A1-Snail mutant	5′CCA ATGA T**TGT**A GGCCT CCC3′
	R: COL1A1-Snail mutant	5′GGG AGGCC T**ACA**A TCAT TGG3′

**Table 4:** Transcriptional factor binding region on candidate Mesenchymal genes.

Mesenchymal Genes Promoter	Proposed Transcription Factors	Proposed Binding Motif	References
FN	Snail	−576 TCACA −570	[36,37]
	E/S overlapping	−360 CGGCGGGCGGGCGGGC −345	[38,39]
MMP9	Snail	−818 TCACA −812	[15,17]
	E/S overlapping	−797 GAGCCCCCCACCCCCC −781	[17]
LEF	Snail	−1287 TCACA −1281	[40,41]
	E/S overlapping	−1195 CCCTCACCCCCCGCCT −1179	
ZEB1	Snail	−1065 TCACA −1059	[17]
	E/S overlapping	−933 AAGAGGGCGGGGAGCG −915	[17]
COX2	Snail	−968 TCACA −962	[42]
	E/S overlapping	−911 CCTTTCCCGCCTCTC −896	
VIMENTIN	Snail	−2318 TCACA −2312	[43]
	E/S overlapping	−1976 CCCCCTGCCGCCACC−1960	[44,45]
VITRONECTIN	Snail	−1373 TCACA −1367	[46,47]
	E/S overlapping	−1238 GCACCCGCCCACCAC −1222	[48]
COL1A1	Snail	−1071 TCACA −1065	[49,50]
	E/S overlapping	−993 CCCCAATCCCCACCTC −976	[51,52]
α-SMA	Snail	−689 TGTGA −683	[31,53]
	E/S overlapping	−507 GCTCTCTCCCCGCCCC −490	[54]
N-cadherin	Snail	−311 TCACA −305	[55,56]
	E/S overlapping	−289 CCCCCGCCCCCTCCCC −272	[57,58]
TWIST1	Snail	−723 TCACA −717	[59]
	E/S overlapping	−606 CCCCGCGCCCGCCGGA −590	

## Data Availability

The datasets during and/or analyzed during the current study are available from the corresponding author on reasonable request.

## Supplementary Materials

The following are available online at https://www.mdpi.com/article/10.3390/cells10092202/s1, Figure S1: Map for ChIP fragment and EMSA probe for FN, LEF, COX2 and COL1A1 promoter, Figure S2: Time course of TPA-induced gene expression of SNA, FN, LEF, COX2, and COL1A1 in HCC340, Figure S3: SNA is essential for constitutive and TPA-induced gene expression of FN, LEF, COX2, and COL1A1, Figure S4: SNA overexpression increased motility of HCC, Figure S5: SNA is required for TPA-induced promoter activation of FN and LEF in HepG2, Figure S6: Promoter sequences of FN, LEF, COX2, and COL1A1 containing Snail and EGR/SP1 overlapping region, Figure S7: Quantitative PCR for ChIP and double ChIP assay for TPA induced binding of transcription factors on promoters of FN and LEF in HCC340.

## Abbreviations

SNA: Snail; MMP9: matrix metalloproteinases 9; ZEB1: zinc finger E-box binding homeobox 1; TPA: the phorbol ester tumor promoter 12-O-tetradecanoyl-phorbol 13-acetate; EGR1: Early Growth Response 1; SP1: proximal specificity protein 1; FN: fibronectin and LEF: lymphoid enhancer-binding factor; COX2: cyclooxygenase 2; COL1A1: collagen type I alpha 1 chain; FOXA2: forkhead box protein A2; PM: point mutation; ChIP: chromatin immunoprecipitation; EMSA: electrophoresis mobility shift assay; EMT: epithelial mesenchymal transition; HCC: hepatocellular carcinoma.
