# Supplementary material for: Snail Upregulates Transcription of FN, LEF, COX2, and COL1A1 in Hepatocellular Carcinoma: A General Model Established for Snail to Transactivate Mesenchymal Genes
Source: Cells. 2021 Aug 26;10(9):2202. doi: 10.3390/cells10092202 (PMC8467536; doi:10.3390/cells10092202)
Supplement: Supplementary file 1 [file cells-10-02202-s001.zip › cells-1323920-supplementary.pdf]

cells

MDPI

Article

Snail Upregulates Transcription of FN, LEF, COX2, and COL1A1 in Hepatocellular Carcinoma: A General Model Established for Snail to Transactivate Mesenchymal Genes

Tam Minh Ly [1], Yen-Cheng Chen [2], Ming-Che Lee [3], Chi-Tan Hu [4], Chuan Chu Chen [5], Hsin-Hou Chang [6], Ren-In You [5,*] and Wen-Sheng Wu [2,*]

Supplemental data

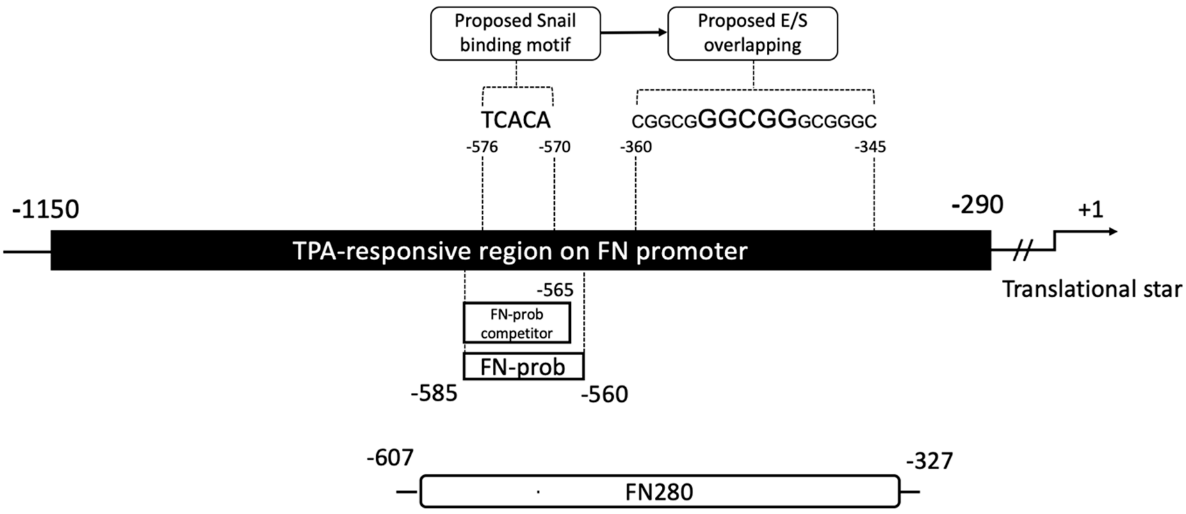

(a)

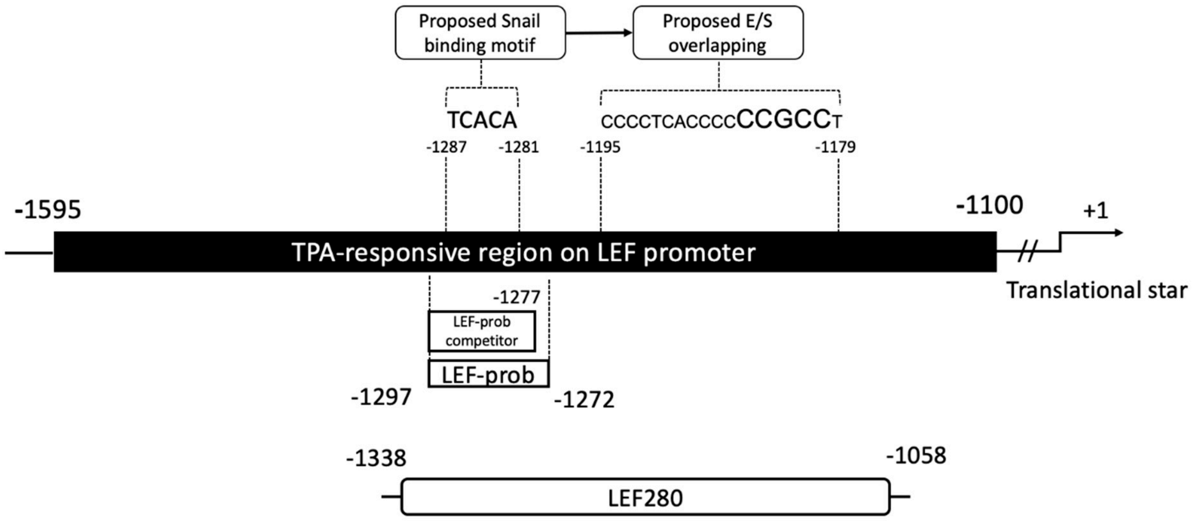

(b)

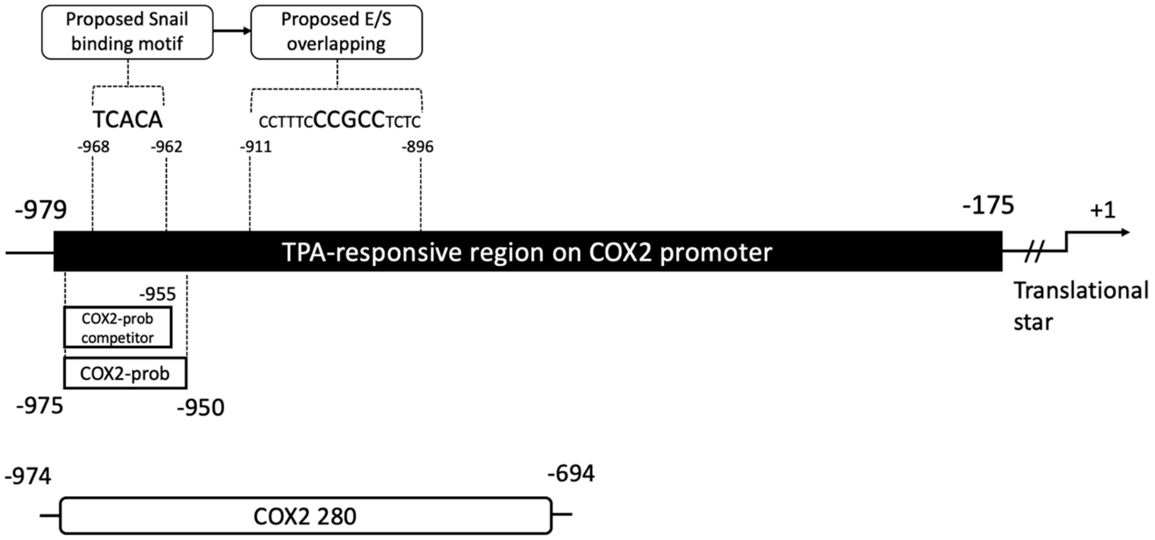

(**c**)

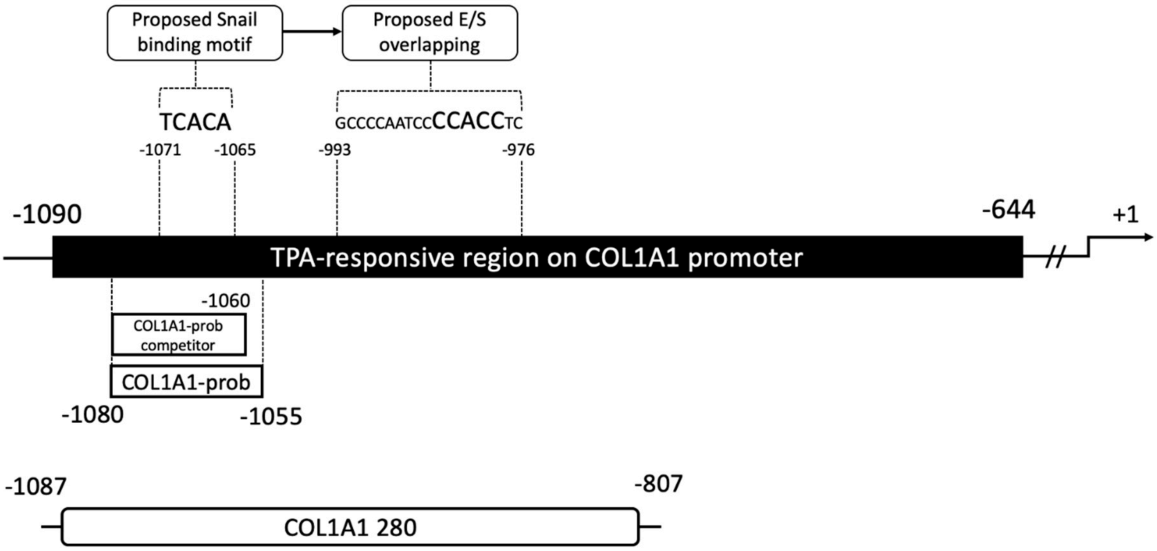

(**d**)

Supplemental Figure S1. Map for ChIP fragment and EMSA probe for FN, LEF, COX2 and COL1A1 promoter**.** Schematic MAP showing the PCR fragments amplified for the ChIP assay of SNA, EGR1/SP1 overlapping; and EMSA probe on FN (**a**), LEF (**b**), COX2 (**c**) and COL1A1 (**d**) promoters.

(**a**)

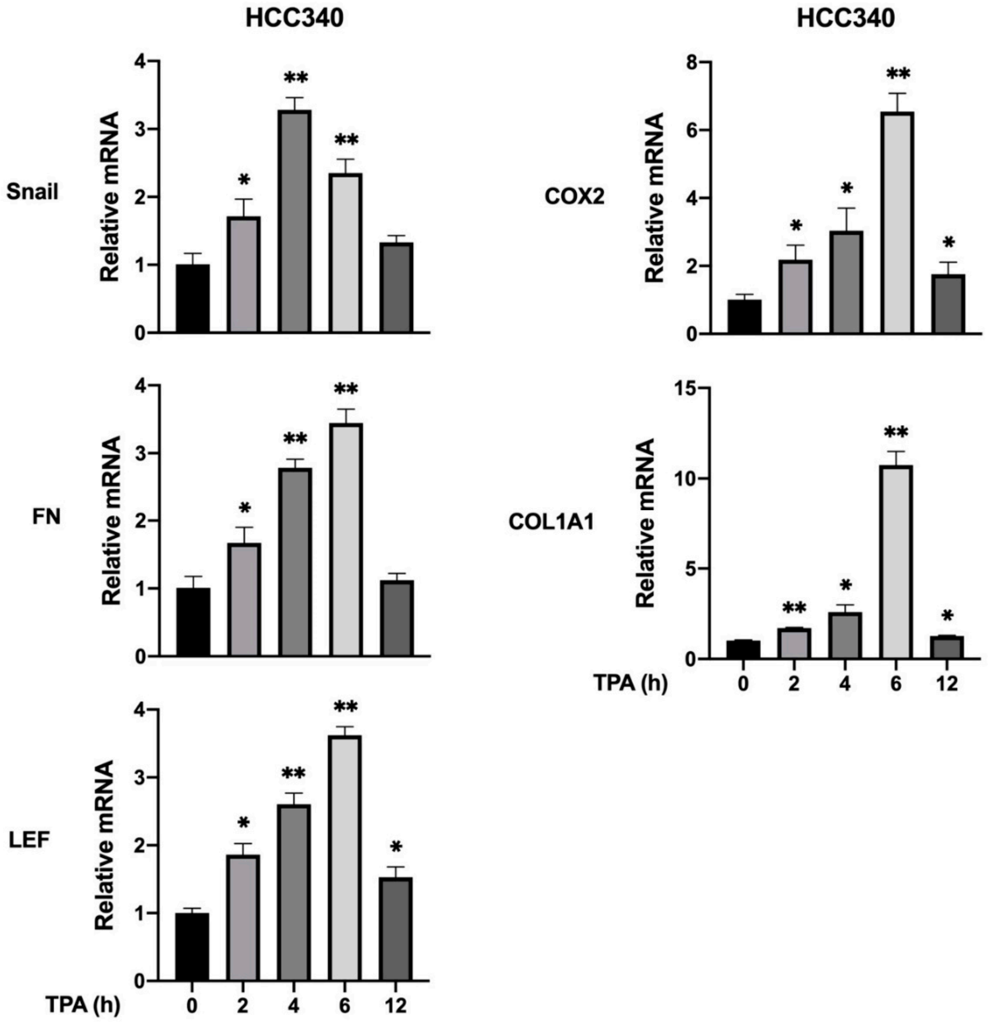
HCC340
HCC340
Snail
COX2
FN
COL1A1
LEF
Relative mRNA
TPA (h)
0
2
4
6
12

(**b**)

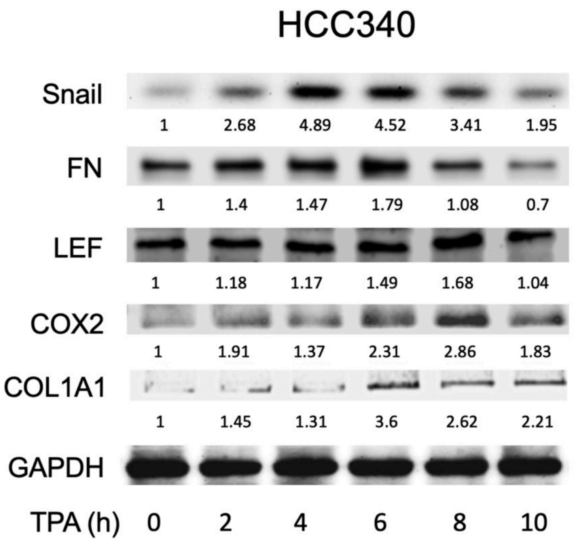
HCC340
Snail
1 2.68 4.89 4.52 3.41 1.95
FN
1 1.4 1.47 1.79 1.08 0.7
LEF
1 1.18 1.17 1.49 1.68 1.04
COX2
1 1.91 1.37 2.31 2.86 1.83
COL1A1
1 1.45 1.31 3.6 2.62 2.21
GAPDH
TPA (h) 0 2 4 6 8 10

Supplemental Figure S2. Time course of TPA-induced gene expression of SNA, FN, LEF, COX2, and COL1A1 in HCC340. HCC340 cells were treated with 50 nM TPA for indicated time (**a**, **b**) Q-RT/PCRs (**a**) and Western blot (**b**) of Table 0. $p < 0.01$, N=3) between the indicated samples and time zero. In (b), the numbers indicated below each band are averaged relative intensities (Coefficient of Variation: 7-10%) taking time zero as 1.0.

(**a**)

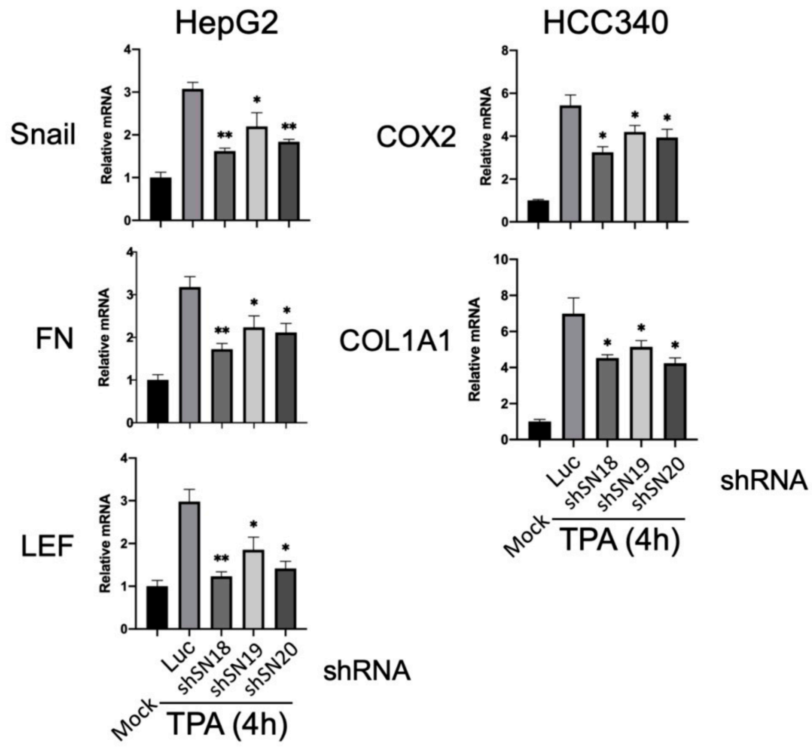

(**b**)

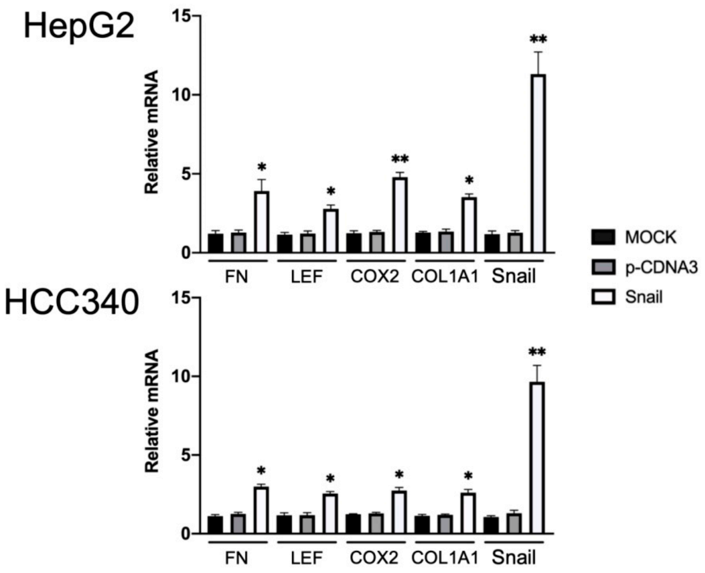

(**c**)

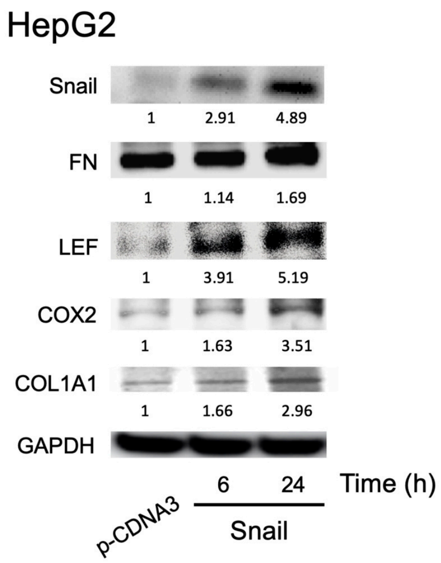

Supplemental Figure S3. SNA is essential for constitutive and TPA-induced gene expression of FN, LEF, COX2, and COL1A1. HepG2 and HCC340 cells were transfected with luciferase (Luc) shRNA or three different shRNA of SNA for 24 h followed by TPA treatment for 4 h (**a**); HepG2 and HCC340 were transfected with SNA expressing

plasmid for 24h (**b**) or indicated time (**c**), using p-CDNA3 as a control vector. Q-RT/PCRs (a), (b) and Western blot (c) of the indicated genes were performed using GAPDH as an internal control. In (a) and (b), (*, **) represent the statistically significant difference ($p < 0.05$, $p < 0.01$, N=3) between the indicated samples and Luciferase shRNA (a) or p-cDNA3 group (b). The data in (c) are representative of three reproducible results. The numbers indicated below each band are averaged relative intensities (Coefficient of Variation: 7-10%) taking p-cDNA3 group as 1.0.

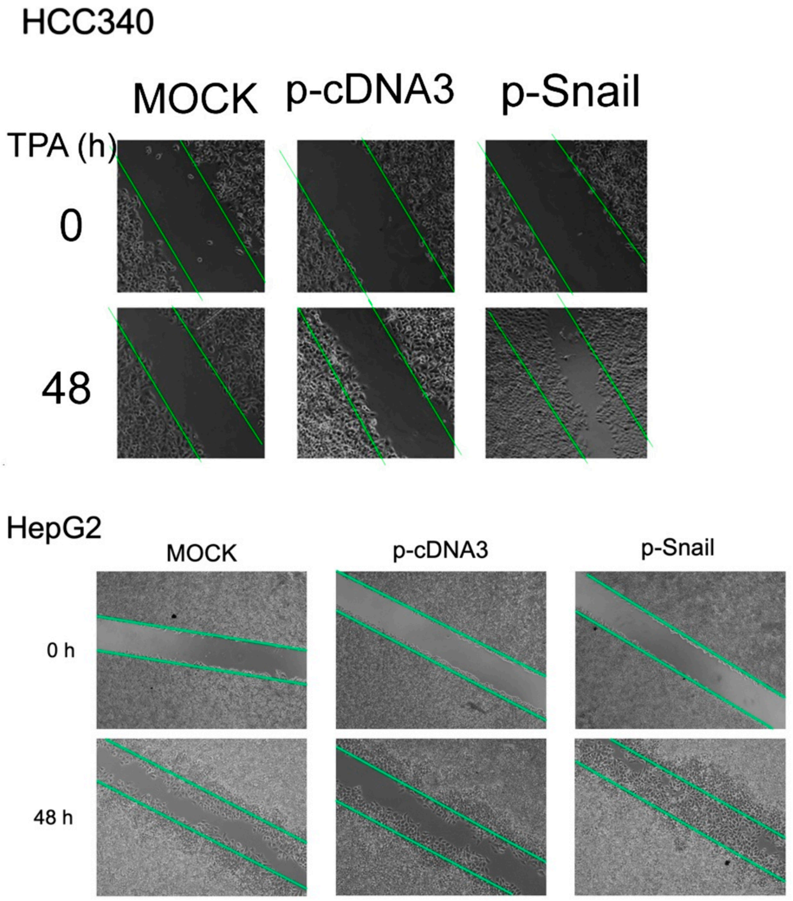

Supplemental Figure S4. SNA overexpression increased motility of HCC340. HCC340 (upper panel) and HepG2 (lower panel) were untransfected (MOCK), transfected with Snail expression plasmid (p-Snail) or control vector (p-cDNA3) for 48 h followed by wound healing assay for 48 h. Pictures were taken at 0 and 24 h after the cells begin to move into the wound area between green lines under serum free condition. Motility of the cell were compared by the difference of the cell migrated into the wound area (indicated by green arrow head).

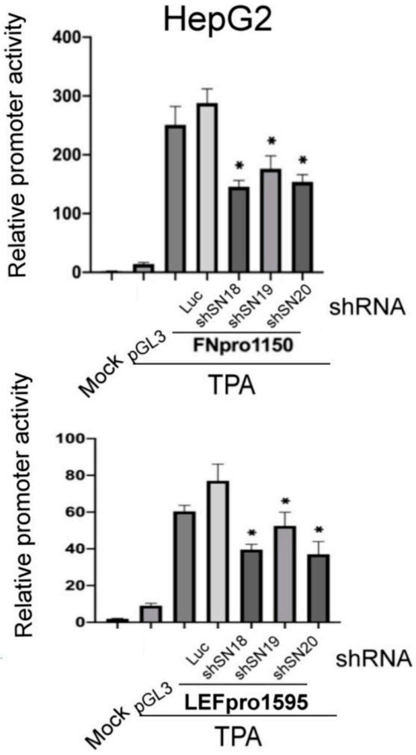

Supplemental Figure S5. SNA is required for TPA-induced promoter activation of FN and LEF in HepG2. HepG2 cells were untransfected (MOCK), transfected with none or various shRNA as indicated for 24 h. Then, the cells were transfected with full-length promoter of FN (upper panel) or LEF (lower panel) for 16 h, followed by treatment with none (MOCK) or TPA for 12 h. Dual luciferase was performed. Relative luciferase activity was calculated, taking the data of MOCK as 1.0. (*, **) represent the statistically significant difference ($p < 0.05$, $p < 0.001$, N = 3) between the indicated samples and Luc shRNA control group.

a. Homo sapiens fibronectin 1 (FN1), RefSeqGene on chromosome 2, NCBI Reference Sequence: NG_012196.1

```
                1150            SN1
CTGGTCCAACTTTCCA/ATTTTCAGGTTGTTCACAGTGATTTCACTTTCAGTGGAAACGGGCGTCCCATC
CCCAGGAAAGGAAGGCTTTCCTGCATGTGTAAAAAAGTAAACTGTTACTTTGTCCTTGCAAAAGAAAACT
TCATTCTCTGAACTTCCCCGGGATCTGCAAAGCGCCCCGCGGAACTCCCGGTACTTAGTAGAAGCTCATT
AAAGGTCTCTGTTCCCCTTTGCTCCCCGTTCGCCCGCTGGCCCTTTCTCAGAGCCAGACAGGCACAGCGC
TGAGAAGGGAAGAAGTCCGAACAGGGAGCTGTGAAGACAAAATAAGGGAGTCCCGAGTCAGTACCCTTTA
                                                                     750
GTCCAAAGAAAGGGAGCGGATGGGGGAAAGGCAGCCCCGCCCTGGGACTGAAAAGTCTGGATTCT/TAA
                                        SN2       700
CAGCTGCAAGGTCGTGGATATTTTATGGGTTTTCTTCCTCACAAAAT/ACACTCCTATAAGCAGAGATTCC
CCCCCTCCACCCCGAAGAGAGGTGACGCAATGTCCTCAAACACTACCACCACCCCCAATAAAAAAGAAAA
                                  SN3     568
GGGAAGGGGGAGCGTCTTGCAACCCCTTCGCTTCACACA/AGTCCAGCCACTCCCTTTCCTCCCAGCCGCT
TCCCATCCCTTCCCCCATCCCCTAAAAAGTTTGATGACCGCAAAGGAAACCGAAAAAAAGTTGTCTTGCC
CCAGTCCTGGCGGGCCATCAGCATCTCTTTTGTTCGCTGCGAACCCACAGTCCCCCGTGACGTCACCCGG
                                       E/S overlapping               330
AGCCCGGGCCAATCGGCGCGCGGTCGGCTGCGGCGGCCGGCGGGCGGGCGGGCGGGTGGGGTGGGGC/GGG
                                        290
GCGGGGACAGCCCGGCGGGTCTCTCCTCCCCCGCGCC/CCGGGCCTCCAGAGGGGCGGGAGGGGACCGTCC
…………………………………………ACAGCGGTGCCCTCCACGGGAGCCTCGAAGAGCAAGAGGCAGGCTCAGCAAATG
```

b. Homo sapiens lymphoid enhancer binding factor 1 (**LEF1**), RefSeqGene on chromosome 4 NCBI Reference Sequence: NG_015798.1.

```
                   1595               SN1
TCTCCCCACTGTCAGAGCATCT/ATCAATGTGGTGTCCATCACAGCGGCAGCGGCTTTCTCTTTCATCTT
CCTCCCTCTGCCAGAGCCAGGGAGGGAGAGTGGAGGCGTCAAGGAGGTAGGGGAGAGACTGGCAGAGGA
AAAGGAGTGGGTGGGTGGGGGCCAAGTAAATAGATACTTAGATGATGAAGTCAAGCCACTGCGGCAATGT
TTCTTGTCAGTTTCACGCGGGCAAAGCGTGCCTTTCGGTGGGTTATAAGCAGCGCCCGGTCCTTCCTTCT
                                       1300         SN2
CTCGCCAAGTTGCCTGATCCTTCCCTCCAGGCGCGCGC/GCACACACCACACTCACACACCCCAAAACCAA
                                      1231
GACTCGTCCTACAGGATCTGGGAAAAGAAAAAGAAAA/AAAAGCCCTCAATCACCACCTCCTTCTCGCCGA
   E/S overlapping 1                              E/S overlapping 2
CTCCCCCTCACCCCCCGCCTCCCCTCCAGCGGCAGCCAAGGAGAGCTAGAGGCGGGGGAGGGGAGAGGG
                  1100
AGGAGAAGCGACGCAA/GTGGGTAGCTTTTCAGCGCCGGCGAGGCGCGGGAGGAGGAGAAGCAGTGGGGAG
........................................AGCGGAGCGGAGATTACAGAGCCGCCGGGATG
```

c. Homo sapiens prostaglandin endoperoxide synthase 2 (COX2), RefSeqGene on chromosome 1 NCBI Reference Sequence: NG_028206.2.

```
                              979          SN
TCCATCAGAAGGCAGGAAACTTTATATTGGT/GACCCGTGGAGCTCACATTAACTATTTACAGGGTAACT
                      921      E/S overlapping
GCTTAGGACCAGTATTATGA/GGAGAATTTACCTTTCCCGCCTCTCTTTCCAAGAAACAAGGAGGGGGTGA
AGGTACGGAGAACAGTATTTCTTCTGTTGAAAGCAACTTAGCTACAAAGATAAATTACAGCTATGTACAC
TGAAGGTAGCTATTTCATTCCACAAAATAAGAGTTTTTTAAAAAGCTATGTATGTATGTGCTGCATATAG
AGCAGATATACAGCCTATTAAGCGTCGTCACTAAAACATAAAACATGTCAGCCTTTCTTAACCTTACTCG
CCCCAGTCTGTCCCGACGTGACTTCCTCGACCCTCTAAAGACGTACAGACCAGACACGGCGGCGGCGGCG
GGAGAGGGGATTCCCTGCGCCCCCGGACCTCAGGGCCGCTCAGATTCCTGGAGAGGAAGCCAAGTGTCCT
TCTGCCCTCCCCCGGTATCCCATCCAAGGCGATCAGTCCAGAACTGGCTCTCGGAAGCGCTCGGGCAAAG
ACTGCGAAGAAGAAAAGACATCTGGCGGAAACCTGTGCGCCTGGGGCGGTGGAACTCGGGGAGGAGAGGG
                    362                                          175
AGGGATCAGACAGGAGAGT/GGGGACTA.......................CATGGGCTTGGTTTT/CAGT
CTTATAAAAAGGAAGGTTCTCTCGGTTAGCGACCAATTGTCATACGACTTGCAGTGAGCGTCAGGAGCAC
GTCCAGGAACTCCTCAGCAGCGCCTCCTTCAGCTCCACAGCCAGACGCCCTCAGACAGCAAAGCCTACCC
CCGCGCCGCGCCCTGCCCGCCGCTGCGATG
```

d. Homo sapiens collagen type I alpha 1 chain (COL1A1), RefSeqGene on chromosome 17 NCBI Reference Sequence: NG_007400.1

```
                                               1090               SN
GGGTCTCTAAGCAGCCCCTGGCCACAGCCATGGCAAACAAAACTC/TTCTCTAAGTCACCAATGATCACA
GGCCTCCCACTAAAAATACTTCCCAACTCTGGGGTGGAAGAGTTTGGGGGATGAATTTTTAGGGGATTGC
   E/S overlapping
AAGCCCCAATCCCCACCTCTGTGTCCCTAGAATCCCCCACCCCTACCTTGGCTGCTCCATCACCCAACCA
CCAAAGCTTTCTTCTGCAGAGGCCACCTAGTCATGTTTCTCACCCTGCACCTCAGCCTCCCCACTCCATC
TCTCAATCATGCCTAGGGTTTGGAGGAAGGCATTTGATTCTGTTCTGGAGCACAGCAGAAGAATTGACAT
CCTCAAAATTAAAACTCCCTTGCCTGCACCCCTCCCTCAGATATCTGATTCTTAATGTCTAGAAAGGAAT
CTGTAAATTGTTCCCCAAATATTCCTAAGCTCCATCCCCTAGCCACACCAGAAGACACCCCCAAACAGGC
   644
AC/ATCTTTTTAATTCCCAGCTTCCTCTGTTT.................TGTCTAGGGTCTAGACATG
```

Supplemental Figure S6. Promoter sequences of FN, LEF, COX2, and COL1A1 containing Snail and EGR/SP1 overlapping region. Promoter region of FN (a), LEF (b), COX2 (c), and COL1A1 (d) quoted from gene bank.

(**a**)

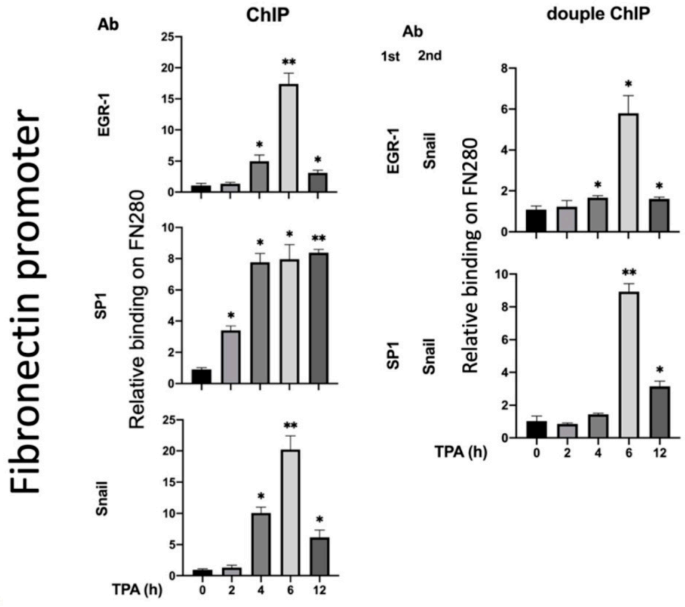

(**b**)

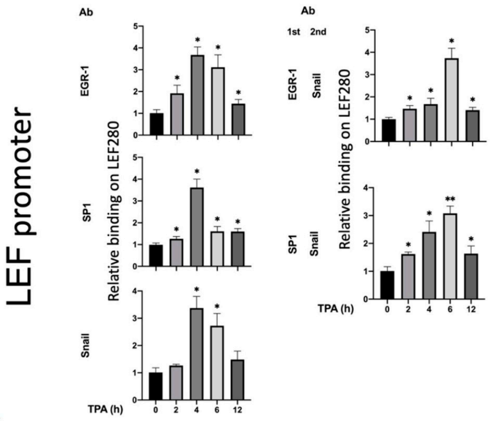

Supplemental Figure S7. Quantitative PCR for ChIP and double ChIP assay for TPA induced binding of Table 340. HCC340 cells were treated with TPA at indicated time. Single ChIP for binding of indicated transcription factor on FN promoter (FN280) (**a**, left panel) and LEF promoter (LEF280) (**b**, left panel), and double ChIP for association of indicated transcriptional factor on FN280 (**a**, right panel) and LEF280 (**b**, right panel) were performed using quantitative PCR. (*, **) represent the statistically significant difference ($p < 0.05$, $p < 0.01$, $N = 3$) between the indicated samples and time zero.
